# Development of a mobile health application to support the management of paediatric FMF

**DOI:** 10.1093/rheumatology/keag288

**Published:** 2026-06-03

**Authors:** Yunus Emre Bayrak, Hikmetcan Özcan, Fatih Pir, Nihal Şahin, Duygu Aydın, Hafize Emine Sönmez

**Affiliations:** Faculty of Medicine, Department of Pediatric Rheumatology, Kocaeli University, Kocaeli, Turkey; Department of Computer Engineering, Kocaeli University, Kocaeli, Turkey; Department of Computer Engineering, Kocaeli University, Kocaeli, Turkey; Faculty of Medicine, Department of Pediatric Rheumatology, Kocaeli University, Kocaeli, Turkey; Faculty of Medicine, Department of Pediatric Rheumatology, Kocaeli University, Kocaeli, Turkey; Faculty of Medicine, Department of Pediatric Rheumatology, Kocaeli University, Kocaeli, Turkey

**Keywords:** FMF, medication adherence, mobile applications, patient monitoring

## Abstract

**Objective:**

FMF is highly prevalent in Mediterranean countries and represents a substantial proportion of paediatric rheumatology patients. Colchicine, the mainstay of treatment, requires lifelong daily use, yet long-term adherence remains challenging, leading to recurrent attacks and increased healthcare burden. This study aimed to develop a freely downloadable mobile application to improve medication adherence by providing reminders and enabling direct communication with physicians.

**Methods:**

The application was designed as a structured medication-timing and monitoring tool, providing time-specific medication reminders, real-time intake confirmation (‘taken/missed’) and digital adherence tracking. The user interface was tailored to the needs of patients and parents, considering children’s attention span, parental workload and health literacy. Following pilot testing in seven volunteer patients, the application was refined based on user feedback. Physicians used a web-based panel to register patients, define prescriptions and treatment schedules, and assign calendar-based appointments. Medication adherence was assessed using the MASIF scale, and disease activity was evaluated using the AIDAI score. A total of 93 patients with FMF were included in the study.

**Results:**

Before application use, adherence was low, with a median MASIF score of 44 and most patients demonstrating poor adherence. After implementation, MASIF scores significantly improved at 3, 6 and 12 months, with a marked reduction in poorly adherent patients (*P* < 0.001). AIDAI scores and attack frequency significantly decreased, and no patient had an AIDAI score >9 during follow-up. Agreement between application-measured adherence and MASIF scores was excellent (ICC > 0.90).

**Conclusion:**

Mobile reminder applications are low-cost, accessible digital health tools with strong potential to enhance medication adherence in FMF patients.

*Rheumatology* key messages

- A disease-specific mobile application significantly improved colchicine adherence in paediatric FMF.
- Improved adherence was associated with reduced disease activity over 12 months.
- Digital adherence monitoring enabled objective, real-time assessment of treatment behaviour.

## Introduction

Familial Mediterranean fever (FMF) is a self-limited autoinflammatory disease characterized by recurrent episodes of fever and serositis, including peritonitis, pleuritis, pericarditis and synovitis [1, 2]. It is highly prevalent in countries surrounding the Mediterranean basin and represents a substantial proportion of patients followed in paediatric rheumatology clinics in Turkey [3]. Due to its chronic nature, FMF requires lifelong daily treatment, and medication adherence plays a critical role in achieving disease control and preventing long-term complications. Clinically, FMF is characterized by recurrent inflammatory attacks accompanied by fever and pain affecting one or more serosal surfaces. These attacks occur at irregular intervals and usually last between 12 h and 3 days [4]. Fever is commonly accompanied by additional symptoms; however, isolated febrile episodes may also occur [5].

Colchicine has been the cornerstone of FMF treatment since 1972 and has markedly reduced the incidence of amyloidosis, one of the most serious complications of the disease [6, 7]. About ∼5–15% of patients continue to experience attacks despite colchicine therapy [8]. Nevertheless, before classifying patients as having colchicine-resistant FMF, medication adherence should be carefully evaluated. Long-term daily colchicine use poses significant challenges, particularly in paediatric and adolescent populations. Previous studies from Turkey have demonstrated that medication adherence decreases with increasing age among children with FMF, and poor adherence is associated with increased attack frequency, higher rates of emergency department visits and hospitalizations, and reduced quality of life [9, 10]. Moreover, insufficient disease control due to non-adherence increases the long-term risk of renal amyloidosis. The most common causes of medication non-adherence are unintentional factors such as forgetfulness and confusion regarding dosing schedules [11]. Simple interventions, including pill organizers, have been shown to improve adherence; however, these strategies may be insufficient in the context of modern lifestyles [12]. Mobile health (mHealth) solutions offer a promising approach to improving adherence, enabling symptom tracking and facilitating patient–physician communication.

In this context, we developed a free, mobile device-compatible application designed specifically for patients with FMF. The application provides daily reminders for medication dosing and timing, enables patients to record clinical symptoms such as fever, abdominal pain, chest pain, musculoskeletal complaints, gastrointestinal symptoms and skin manifestations, and allows real-time communication of disease- or treatment-related concerns with the treating physician. By facilitating systematic and timely symptom recording, this tool aims to enable a more accurate assessment of disease activity and severity and to support optimized disease management in paediatric FMF.

## Materials and methods

This study was designed as a feasibility pilot study to evaluate the usability, acceptability and preliminary effectiveness of mHealth application developed to support medication adherence in paediatric FMF patients.

### Study design and system overview

A multi-layer digital adherence system was developed to support medication adherence monitoring in paediatric patients. The system comprised (i) a native mobile application (Android and iOS) for patients/parents and (ii) a web-based physician dashboard for patient registration, prescription entry, treatment scheduling and appointment planning. User-centred design principles were applied by considering children’s attention span and parents’ daily routines and health literacy.

### Implementation and architecture

The backend was implemented in Java using the Spring Boot framework and exposed RESTful APIs for secure client–server communication. Data were stored in a MySQL relational database using normalized schemas covering patient records, prescriptions, medication schedules, appointment calendars, notification responses, symptom entries and adverse-event reports. Passwords were stored as cryptographic hashes (SHA-256). Role-based access control was implemented to restrict access to authorized physicians, and all data transfer was performed over HTTPS.

The backend system was deployed on secure on-premises servers located in the university’s data centre. All patient data were stored within the university’s internal network infrastructure to ensure institutional control over data governance and compliance with national data protection regulations.

The web interface was developed using Next.js (React-based) with Bootstrap components and designed responsively for desktop and tablet use. Mobile applications consumed the REST APIs and provided medication reminders, intake confirmation (‘taken/missed’), symptom tracking, adverse-event reporting and secure messaging.

### Data synchronization

To maintain data consistency, two synchronization mechanisms were used: (1) scheduled background tasks running every 4 h to fetch updated prescription and appointment data and (2) real-time synchronization triggered on in-app page transitions following an internet connectivity check.

### Pilot testing and refinement

The mobile application underwent pilot testing in seven volunteer patients. Feedback obtained during this pre-test phase informed iterative refinements to notification flow, button placement and wording before final deployment.

### Participants and procedures

Patients were eligible if they (i) had been followed with a diagnosis of FMF for ≥1 year, (ii) were >12 years of age, (iii) had no comorbidities and (iv) owned a smartphone. All patients fulfilled the Eurofever/PRINTO classification criteria for FMF [13].

During outpatient clinic visits, after the mobile application link was shared, healthcare staff installed the application on participants’ devices. The application used on the patient side was publicly available on Google Play (https://play.google.com/store/apps/details?id=com.koru.romatoloji&hl=tr), and physicians accessed the secure web-based panel at http://koru.kocaeli.edu.tr:3000/. Access to the web-based panel was restricted to authorized physician personnel only; it enabled patient registration, prescription entry, appointment scheduling, survey assignment and the review of adverse event and notification data.

**Figure 1:**
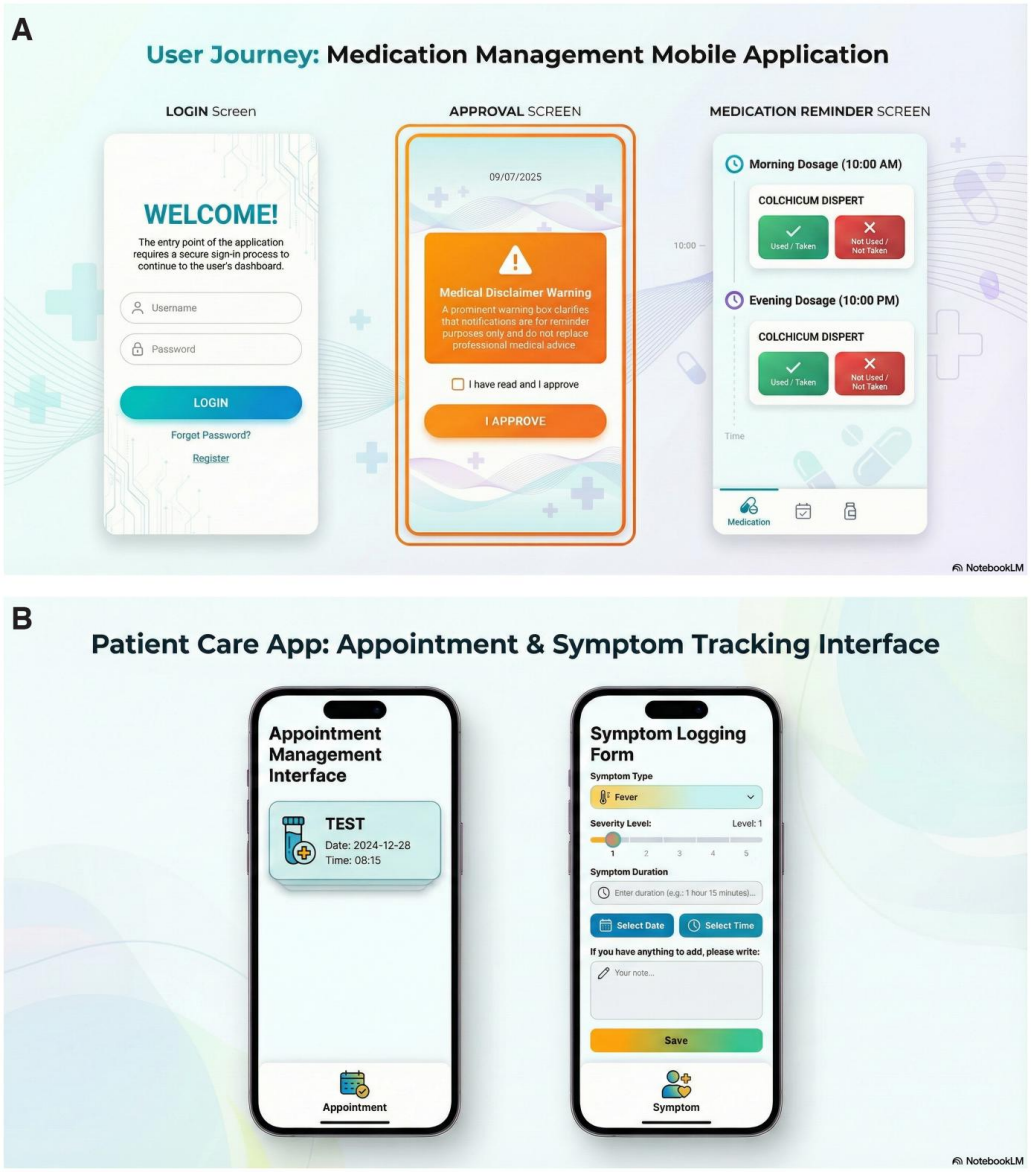
Representative images of the core interfaces of the mobile application. (A) Medication management module showing the login screen, approval screen, and medication reminder interface used to support colchicine adherence. (B) Patient care module displaying the appointment management interface and symptom logging form for tracking appointments and disease-related symptoms

Following installation, parents/caregivers were provided with standardized face-to-face training lasting ∼15 min. This training covered the prescription-tracking screen, appointment screen, adverse-event reporting screen and notification-management functions. During the same outpatient visit, patient registration and prescription entry were completed, enabling immediate login to the application and its use. Additional technical support was provided when needed during the follow-up period. Participants were instructed to confirm each reminder by selecting ‘taken’ or ‘missed’, to record symptoms and their duration as needed, and to submit medication-related questions via in-app messaging (Fig. 1A and B).

During follow-up, one participant was excluded from the study due to the discontinuation of their treatment.

### Outcomes and follow-up

Participants provided paper-based Autoinflammatory Disease Activity Index (AIDAI) records for the preceding 3 months [14], and adherence was evaluated using the MASIF (Medication Adherence Scale for FMF) [15] to compare self-reported measures with app-based real-time tracking. The MASIF score ranges from 18 to 90 and consists of 18 items, each evaluated on a 5-point Likert scale (1–5); scores ≥60 indicate good medication adherence, whereas scores <60 indicate poor adherence. Both scores were routinely administered to patients with FMF in routine clinical practice. After the initiation of the study, the MASIF questionnaire was administered to patients through the application every 3 months. Patients were also able to complete the AIDAI forms via the application daily whenever they experienced symptoms. Application-derived adherence and symptom data were logged and made available to physicians through the dashboard for objective monitoring. Study outcomes were assessed at months 3, 6 and 12 after enrolment.

A total of 93 patients aged >12 years were included in the study. Data used for research outputs were analysed in anonymized form in accordance with applicable data protection regulations and ethics approval (GOKAEK-2023/14.19).

### Statistical analysis

Statistical analyses were performed using SPSS (version 22; IBM Corp., Armonk, NY, USA). Continuous variables were tested for normality using the Shapiro–Wilk test and visual inspection of histograms and *Q*–*Q* plots. Normally distributed variables were expressed as mean ± S.D., whereas non-normally distributed variables were reported as median, minimum and maximum. Categorical variables were summarized as frequencies and percentages. Medication adherence derived from the mobile application was calculated as the percentage of scheduled doses confirmed as ‘taken’ over the observation period. Changes in adherence rates and disease activity measures over time (baseline, 3rd, 6th and 12th months) were analysed using Friedman test for non-parametric data. *Post hoc* pairwise comparisons were performed with Bonferroni correction where appropriate. Changes in the proportions of patients with AIDAI scores >9 and MASIF scores <60 across baseline, 3, 6 and 12 months were analysed using Cochran’s *Q* test for related samples. Agreement between app-based adherence scores and MASIF scores was assessed using a two-way mixed-effects intraclass correlation coefficient (absolute agreement). A *P-*value <0.05 was considered statistically significant. *A priori* sample size calculation was performed using G*Power software, indicating that a minimum of 75 participants would be required to achieve adequate statistical power (power = 80%, *α* = 0.05).

### Ethics

The study followed the Declaration of Helsinki guidelines and was approved by the local Ethics Committee (GOKAEK-2023/14.19). Written informed consent was obtained from the patients and their parents.

## Results

### Study population

A total of 93 patients were included in the study. Demographic and clinical findings and treatments are summarized in Table 1. The most frequent mutation was M694V/– (*n* = 40), followed by E148Q/– (*n* = 11) and M694V/M694V (*n* = 9). Compound heterozygous genotypes such as M694V/M680I (*n* = 7) and M694V/V726A (*n* = 4) were also observed. Other variants, including V726A/–, M680I/– and M694V/E148Q, were less common. Rare genotypes (each *n* = 1) comprised R761H/–, M694V/R408Q, M694I/–, K695R/–, E251K/K695R, E148Q/E148Q and others. Overall, M694V was the predominant allele in this cohort, either in heterozygous, homozygous or compound heterozygous combinations (Supplementary Table S1). No treatment modifications, including dose adjustments or additional therapeutic interventions, were made during the study period. All patients continued their baseline colchicine therapy without change.

**Table 1:** The demographic and clinical findings and treatments of the patients.

Demographic findings	
Sex (female/male)	45/48
Age, year, median (minimum–maximum)	12 (12–18)
Age at symptom onset, year, median (minimum–maximum)	4 (1–15)
Age at diagnosis, year, median (minimum–maximum)	5.5 (1–15.5)
Duration of follow-up, month, median (minimum–maximum)	45.5 (12–163)
Family history of FMF, *n* (%)	41 (44.1)
Family history of amyloidosis, *n* (%)	2 (2.2)
**Clinical findings, *n* (%)**	
Fever	80 (86)
Abdominal pain	79 (84.9)
Chest pain	14 (15)
Arthritis	21 (22.6)
Arthralgia	48 (51.6)
Erysipelas-like erythema	3 (3.2)
Attack duration, day, median (minimum–maximum)	3 (1–4)
**Treatment, *n* (%)**	
Resistant to colchicine treatment	2 (2.2)
Biological therapy	2 (2.2)

### Characteristics of the study group before and after mobile application implementation

Among the 93 patients, 36 (38.7%) were self-administering their medication, whereas in 57 patients (61.3%) the medication was administered by their mothers. During the 3 months preceding the intervention, 32 of the 93 patients (34.5%) reported experiencing at least one attack. In this pre-intervention period, the median number of attack days was 2 (0–3), and the median number of emergency department visits was also 2 (0–3). During attacks, the laboratory findings were as follows: the median white blood cell count was 13 680/mm^3^ (4430–19 150), haemoglobin 12.2 g/dl (9.6–14.4), platelet count 308 000/mm^3^ (197 000–579 000), CRP 64.6 mg/l (11.7–320.7) and ESR 28 mm/h (10–86). Before the intervention, the median MASIF score was 44 (34–74). Fifty-nine (63.4%) patients had MASIF scores below 60, indicating poor medication adherence, whereas the remaining 34 (36.6%) patients had scores ≥60, reflecting good adherence. The median AIDAI score was 2 (0–26), and 20 (21.5%) patients had AIDAI scores >9.

**Figure 2:**
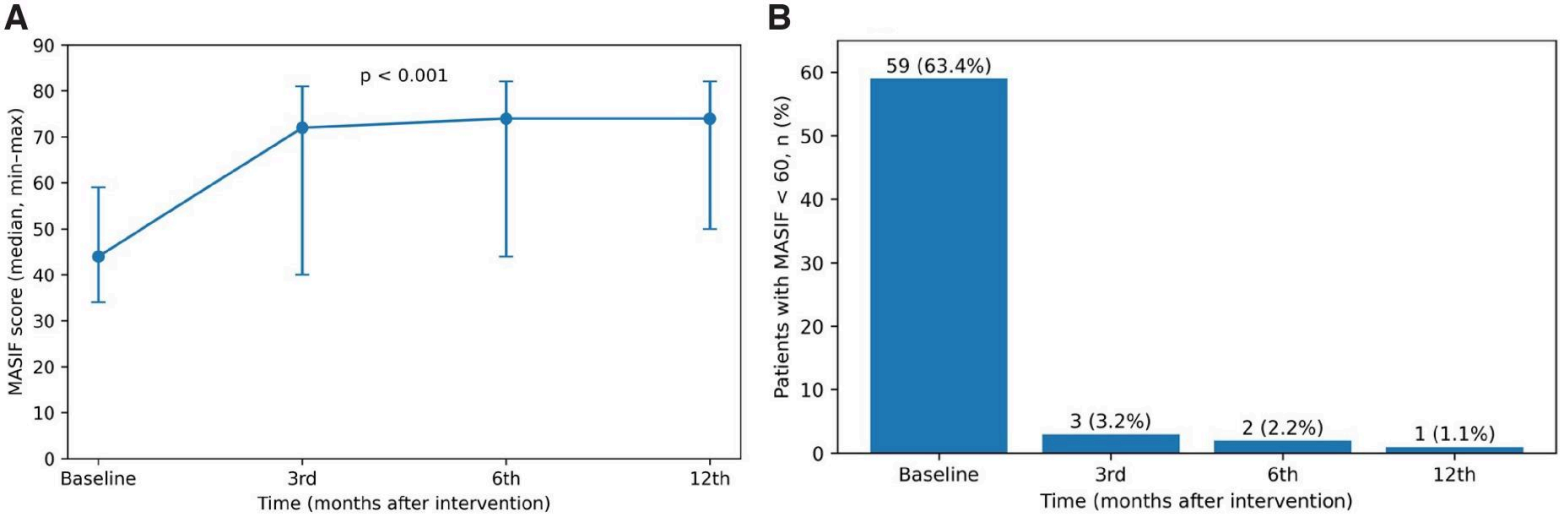
Changes in medication adherence over time following the intervention. (**A**) Median MASIF scores (with minimum–maximum) at baseline and at 3, 6 and 12 months after the intervention. MASIF scores increased significantly compared with the 3-month pre-intervention baseline period (*P* < 0.001). (**B**) Number of patients with poor medication adherence (MASIF < 60) at each time point, demonstrating a marked reduction after the intervention

At the third month after the intervention, the median MASIF score was 72 (40–81), and only three patients had scores below 60. At the sixth month, the median MASIF score increased to 74 (44–82), with only two patients scoring below 60. By the 12th month, the median score was 74 (50–82), and only one patient had a score below 60. Compared with the baseline assessment conducted during the 3 months prior to the intervention, MASIF scores showed a statistically significant increase following the implementation of the application (*P* < 0.001) (Fig. 2A and B).

**Figure 3:**
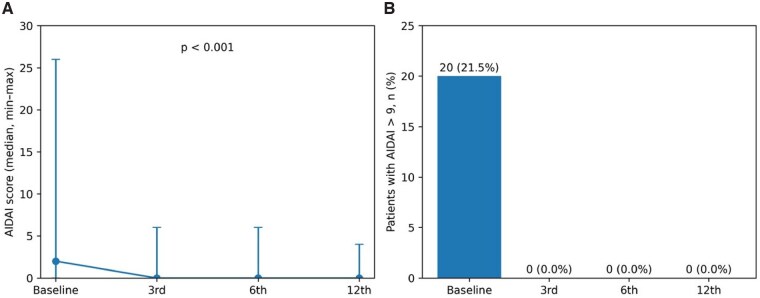
Changes in disease activity over time. (**A**) Median AIDAI scores (with minimum–maximum) at baseline and at 3, 6 and 12 months following the intervention. (**B**) Number and percentage of patients with AIDAI scores >9 at each time point. A significant reduction in the proportion of patients with AIDAI >9 was observed compared with baseline (*P* < 0.001)

At the third month following the intervention, seven patients reported experiencing an attack. Evaluation of attack characteristics revealed that fever was present in all cases; three patients reported chest pain, three nausea and vomiting, two abdominal pain and one joint pain. At the sixth month, three patients reported an attack, all characterized by fever and abdominal pain. Similarly, at the 12th month, three patients experienced an attack, with fever and abdominal pain present in all cases. During both follow-up assessments, the median number of attack days was 0 (0–1), and the median number of emergency department visits was also 0 (0–1). The median AIDAI score was 0 (0–6) at the third month, 0 (0–6) at the sixth month and 0 (0–4) at the 12th month. No patient had an AIDAI score >9 at any follow-up time point (*P* < 0.001) (Fig. 3).

Patients received daily reminders through the application to take their medication and were asked to confirm whether they had taken it. Agreement between app-recorded adherence data and MASIF scores collected every 3 months via questionnaire was excellent, with an intraclass correlation coefficient (ICC) >0.90. The median time to provide the ‘taken’ confirmation (timestamp) was 23.5 min (min–max: 0–113). Sixty patients provided ‘taken’ confirmation within 30 min of the scheduled dose time, whereas the remaining patients confirmed intake between 30 and 120 min.

Only five patients included in the study reported dissatisfaction with the application. The reasons for dissatisfaction were related to system error messages and delays in resolving technical issues.

## Discussion

In the present study, use of the mobile application was associated with a statistically significant increase in MASIF scores compared with the pre-intervention period (*P* < 0.001), indicating improved treatment adherence. Consistent with this improvement, a concomitant reduction in disease activity scores was observed. Moreover, the proportion of patients with an AIDAI score >9 decreased significantly following mobile application use (*P* < 0.001), supporting the clinical relevance of enhanced adherence facilitated by digital health interventions.

Ensuring optimal treatment adherence is a cornerstone of effective chronic disease management, with direct implications for clinical outcomes and health-related quality of life. Medication non-adherence remains a pervasive challenge, particularly in conditions requiring long-term therapy, and is a major determinant of suboptimal disease control. The World Health Organization reports that adherence rates in chronic diseases remain ∼50% even in high-income countries [16]. Forgetfulness, diminished treatment-related motivation, regimen complexity and medication-related adverse effects are consistently identified as key contributors to non-adherence.

The rapid expansion of mHealth technologies has introduced scalable and patient-centred strategies to address medication non-adherence. Smartphone-based medication reminder applications provide time-specific prompts that support regular drug intake and reduce unintentional omissions. Beyond simple reminders, advanced applications enable longitudinal recording of medication use, symptoms and disease activity, thereby facilitating patient self-management and data sharing with healthcare professionals. Accumulating evidence suggests that mHealth-based reminder interventions can significantly improve medication adherence, with particularly robust effects in younger populations and in chronic diseases requiring polypharmacy. Earlier mobile phone-based interventions, including short message service (SMS) reminders and telephone calls, have demonstrated efficacy in promoting adherence [17]. The widespread penetration of smartphones has further expanded the potential of mHealth solutions, enabling the development of interactive applications that integrate disease monitoring with clinical care [18]. A recent systematic review evaluating mHealth interventions in adolescents and young adults with chronic diseases has shown that these tools are generally feasible, acceptable and easy to use, with common features including medication reminders, symptom tracking and communication with healthcare providers. However, despite high initial acceptability, user engagement tends to decline over time, and the overall evidence for clinical effectiveness remains limited. Importantly, variability in user preferences and barriers such as accessibility and usability highlight the need for patient-centred design and disease-specific interventions [19]. Existing mHealth studies have predominantly focused on chronic conditions such as asthma and diabetes, with a notable lack of applications developed within the field of paediatric rheumatology. To our knowledge, this is the first medication reminder application specifically designed for patients with FMF. During the study period, no additional or more frequent clinical interventions were implemented based on digital monitoring data, and patients were followed according to routine clinical practice. Therefore, the observed clinical improvements are more likely attributable to enhanced medication adherence supported by the application’s reminder and real-time monitoring functions, rather than increased clinical intervention.

The 2024 update of the EULAR/PReS recommendations for the management of FMF emphasizes the need for a standardized minimum core outcome set to harmonize clinical care and research [20]. This core set includes objective measures of disease activity (e.g. attack frequency over the preceding 3 months and physician global assessment), patient-reported outcomes and experiences (PROMs and PREMs) and inflammatory biomarkers such as CRP and serum amyloid A (SAA). By allowing daily documentation of attacks, symptoms and medication adherence, the mobile application evaluated in this study operationalizes this recommended core outcome set in routine clinical practice. This digital approach enables clinicians to assess disease activity and treatment response using contemporaneous, patient-generated data while simultaneously capturing patient experience and adherence behaviour. Numerous studies have evaluated medication adherence in children with FMF and have identified several factors associated with non-adherence. Increasing age [9], low health literacy [10] and the presence of erysipelas-like erythema at diagnosis [21] have all been reported to be significantly associated with poor adherence. Although these studies have contributed to understanding the predictors and potential underlying reasons for non-adherence, interventional studies specifically designed to improve medication adherence in FMF are lacking. In this context, our study contributes to existing literature by moving beyond the identification of risk factors and evaluating a structured intervention designed to improve medication adherence in this patient population.

Another important clinical implication of our findings is the potential role of structured and real-time adherence monitoring in distinguishing true colchicine resistance from poor adherence. In routine practice, ongoing attacks despite colchicine therapy may lead to the assumption of colchicine resistance and subsequent escalation to biologic treatments. However, suboptimal adherence is a common and often under-recognized contributor to apparent treatment failure. By providing time-specific reminders, real-time intake confirmation and longitudinal adherence tracking, the application enables objective assessment of medication-taking behaviour. This may support clinicians in identifying patients with inadequate adherence and implementing targeted interventions before labelling them as colchicine-resistant, thereby potentially optimizing treatment decisions and avoiding unnecessary initiation of costly biologic therapies.

This study has several limitations that should be considered when interpreting the findings. First, the lack of a control group and the pre–post design limit causal inference, and the observed improvements may partly reflect increased patient awareness, behavioural changes over time or a Hawthorne effect. Although within-subject comparisons reduce inter-individual variability, results should be interpreted cautiously. In addition, the use of a smartphone-based application requires a certain level of digital literacy, which may have introduced selection bias by underrepresenting patients with limited access to or familiarity with mobile technologies. Adherence and disease activity data were partly patient reported; although real-time entry reduces retrospective errors, reporting and recall bias cannot be fully excluded. Finally, the single-centre design and relatively small sample size may limit the generalizability of the findings. Randomized controlled studies with larger and more diverse populations are needed to confirm the effectiveness of the intervention. Smartphone-based medication reminder applications are low-cost, accessible and easily scalable digital health solutions with the potential to improve medication adherence. However, robust evidence for their long-term effectiveness and sustained impact on patient behaviour remains limited. Because the present study has a 12-month follow-up, further studies with longer follow-up periods and larger populations are warranted to evaluate the durability of adherence improvements and their long-term clinical implications.

## Supplementary Material

keag288_Supplementary_Data

## Data Availability

The datasets generated and/or analysed during the current study are available from the corresponding author upon reasonable request.
