# Supplementary material for: Development of a mobile health application to support the management of paediatric FMF
Source: Rheumatology (Oxford). 2026 Jun 3;65(6):keag288. doi: 10.1093/rheumatology/keag288 (PMC13262799; doi:10.1093/rheumatology/keag288)
Supplement: keag288_Supplementary_Data [file keag288_supplementary_data.docx]

**Supplementary Table S1.** Distribution of MEFV genotypes among patients

| **Genotype** | **N** | **Genotype** | **N** |
| --- | --- | --- | --- |
| M694V/- | 40 | R761H/- | 1 |
| E148Q/- | 11 | P369S/A408G | 1 |
| M694V/M694V | 9 | M694V/R408Q | 1 |
| M694V/M680I | 7 | M694I/- | 1 |
| M694V/V726A | 4 | K695R/- | 1 |
| M680I/- | 4 | K695R/- | 1 |
| V726A/- | 3 | E251K/K695R | 1 |
| M694V/E148Q | 3 | E148Q/E148Q | 1 |
| R761H/V726A | 1 | E148Q/P369S | 1 |
| A744S/- | 1 | E148Q/P190P | 1 |
